# Temporal patterns of tuberculosis-associated hyperglycaemia and its effect on treatment outcomes in individuals not previously diabetic: A prospective cohort study

**DOI:** 10.1371/journal.pone.0358593

**Published:** 2026-09-15

**Authors:** Victor Moses Musyoki, Marianne Mureithi, Annamari Heikinheimo, Elizabeth Maleche-Obimbo, Dishon Maina, Susan Musau, Ruth Maganga, Omu Anzala, Kariuki Njaanake

**Affiliations:** 1 Department of Medical Microbiology and Immunology, University of Nairobi, Kenya; 2 KAVI-Institute of Clinical Research, University of Nairobi, Kenya; 3 Tuberculosis and HIV Co-infection Training Program, Kenya; 4 Department of Veterinary Medicine, University of Helsinki, Finland; 5 Department of Paediatrics and Child Health, University of Nairobi, Kenya; 6 Rhodes Chest Clinic, Nairobi, Kenya; 7 Department of Applied Health Sciences, University of Birmingham, United Kingdom; King Faisal University College of Veterinary Medicine and Animal Resources, SAUDI ARABIA

## Abstract

**Background:**

Active tuberculosis (TB) infection can induce hyperglycaemia and insulin resistance, increasing the risk of type 2 diabetes mellitus and worsening TB disease severity. However, data on the dynamics of TB-associated hyperglycaemia and its effect on TB treatment outcomes among individuals not previously diabetic remain limited, particularly in high TB-burden settings. In this study, we aimed to investigate the prevalence and temporal patterns of TB-associated hyperglycaemia and its impact on disease presentation and TB treatment outcomes among a cohort of individuals not previously diabetic.

**Methods:**

This was a five-month longitudinal prospective cohort study conducted at two urban specialized TB healthcare facilities in Kenya among adults aged >18 years with bacteriologically confirmed pulmonary TB (PTB). Known diabetes mellitus (DM) or prediabetic individuals were excluded during enrolment. Glycaemic status was assessed at baseline and months two and five using glycated haemoglobin (HbA1c) according to the American Diabetes Association guideline. Hyperglycaemia was defined as HbA1c ≥5.7% and categorized as normoglycaemia, persistent, transient, or incident hyperglycaemia based on longitudinal measurements. Socio-demographic and clinical data were collected using a structured questionnaire and analysed using SPSS version 26.0 and GraphPad Prism version 9.0. Longitudinal trends in HbA1c levels and the association of hyperglycaemia with DM-related symptoms, as well as TB treatment outcomes, were assessed using the Friedman test and the Generalized Estimating Equations (GEE), respectively.

**Results:**

We enrolled and followed 87 individuals newly diagnosed with PTB. Of these, 66 were tested for hyperglycaemia in month two after 7 died and 14 were lost to follow-up/transferred out (LTFU/TO), and 70 in month 5, after an additional death, 7 re-engagements, and 2 LTFU/TO were reported. At enrolment, the cohort was predominantly male (76%), not married (53%), and HIV-negative (84%), with a median age of 37 years (IQR 29–44). Prevalence of hyperglycaemia at baseline was 75% (95% Confidence Interval [CI] 65–83), with 45% of the individuals presenting as prediabetic and 30% as diabetic. At the month-two and month-five time points, the proportion of participants with prediabetes (pre-DM) increased from 24% (16/66) to 30% (21/70), while the prevalence of DM declined from 21% (14/66) to 9% (6/70). Across the time points, HbA1c levels varied significantly (p < 0.001). In GEE analysis, hyperglycaemic participants were more likely to experience classic symptoms of DM such as frequent micturition (adjusted Relative Risk [aRR] 1.76, 95% CI 1.12–2.77), increased thirst (aRR 2.37, 95% CI 1.38–4.05), unintentional weight loss (aRR 1.84, 95% CI 1.24–2.75), and blurred vision (aRR 1.95, 95% CI 1.20–3.18). After adjusting for age, BMI, HIV, alcohol use, and smoking, hyperglycaemia was independently associated with an increased risk of sputum positivity (aRR 4.22, 95% CI 2.09–8.52). Glycaemic variability was significantly associated with unfavourable TB treatment outcomes (p < 0.001).

**Conclusions:**

New-onset hyperglycaemia was highly prevalent in adults newly diagnosed with TB and persisted through the fifth month of TB treatment. Hyperglycaemia was associated with classic diabetic symptoms and persisting sputum positivity, highlighting the need to integrate glycaemic monitoring and management into routine TB follow-up care.

## Introduction

Tuberculosis (TB), a highly infectious disease caused by *Mycobacterium tuberculosis* (MTB) bacteria, remains the world’s leading cause of death from a single infectious agent [1,2]. According to the World Health Organization (WHO) Global TB Report 2025, an estimated 10.7 million people developed TB worldwide in 2024, of whom approximately 1.23 million died [2]. Countries such as China, the Philippines, India, Pakistan, Indonesia, Bangladesh, Nigeria, and other sub-Saharan African countries continue to report the highest number of TB cases [2,3].

Despite high TB cases reported in most of the developing countries, treatment using conventional antibiotics has effectively increased the cure rates, although recurrent TB and mortality cases during and after TB treatment have been reported [2,4,5]. Recent literature shows that these cases are partly attributed to the co-existence of TB with other conditions and, lately, with hyperglycaemia as a pathological characteristic of TB disease in non-diabetic individuals [6]. The co-existence of TB with chronic conditions such as diabetes mellitus (DM) and the non-diabetic hyperglycaemic state is attracting global attention due to the probable association with increasing cases of TB morbidity, poor TB treatment outcomes, and T2DM [1,5,6]. Pathophysiologically, infection with MTB disrupts hormonal and cytokine homeostasis, increasing the production of pro-inflammatory cytokines such as tumor necrosis factor-alpha (TNF-α), interleukin-1 (IL-1), and interleukin-6 (IL-6) that impair pancreatic β-cell function and insulin signalling, altering insulin production and activity [7,8]. This initiates a glucose intolerance state, stimulating glucose production by the liver; hence, an increase in blood glucose levels in these patients [7–11]. Similarly, available literature suggests that anti-TB drugs such as rifampicin and isoniazid have a hyperglycaemia-inducing effect, especially during the intensive phase of TB treatment [12,13]. Hyperglycaemia weakens the immune system, creating an imbalance between pro-inflammatory and anti-inflammatory cytokines [10,14]. This condition halts the effective response and control of MTB before and during TB treatment [14]. Studies on TB treatment outcomes have shown that hyperglycaemic patients may experience unfavourable outcomes [1,15,16]. Hyperglycaemia reduces the absorption and metabolism of drugs; hence, the prediction that patients presenting with TB and hyperglycaemia have low concentrations of anti-TB drugs in the blood and tissues and are at high risk of transmitting TB [1,13,15,16].

Information on hyperglycaemia during TB disease and treatment, especially among individuals not previously diabetic, remains limited worldwide, including in low- and middle-income countries where TB is endemic and prevalent [1,7]. Studies from Peru, India, China, South Africa, and Nigeria have reported over 25% prevalence of hyperglycaemia among patients with pulmonary TB [1,7,12,17]. However, the majority of these and similar studies have largely been of cross-sectional design, providing little insight into the dynamics of hyperglycaemia during TB treatment. In the present study, we employed a prospective cohort design to assess the glycaemic changes of TB-associated hyperglycaemia in newly diagnosed TB patients previously not diabetic and its impact on TB treatment outcome. The study aimed to generate new evidence to inform future clinical management strategies, address a key gap in the current literature, and improve understanding of the effects of metabolic response to TB infection and treatment.

## Methods

### Study design and population

This was a five-month longitudinal prospective cohort study conducted between February 6, 2024, and November 20, 2024. We recruited newly diagnosed individuals with active PTB who presented for care at Mbagathi County Hospital and Rhodes Chest Clinic, TB-specialized public healthcare facilities in Nairobi, Kenya. We included individuals who were above the age of 18 years with clinical symptoms of PTB, mainly persistent cough lasting more than 2 weeks, fever, chest pain, night sweats, and fatigue, and who were medically confirmed using sputum specimens tested with the Xpert^®^ MTB/RIF Ultra (Cepheid, Sunnyvale, CA, USA) molecular assay according to the manufacturer’s instructions. We excluded individuals who were pregnant, lactating, known pre-DM or DM, and those presenting with multidrug-resistant (MDR-TB) or rifampicin-resistant TB (RR-TB). We also excluded participants who had other chronic diseases, such as cancer or cardiovascular diseases. All the participants were followed for 5 months and interviewed at baseline (prior to TB treatment initiation) and two and five months after starting TB treatment. At each visit, clinical examinations were performed to assess vital signs, the presence of TB-related symptoms, and treatment complications.

### Data, specimen collection, and follow-up TB diagnosis

After consenting and enrolment of the study participants, data were collected using a structured questionnaire at baseline and during the 2^nd^ and 5^th^ month follow-up visits. At baseline, socio-demographic information including age, sex, marital status, education level, smoking, and alcohol use was collected. Weight and height were measured and body mass index (BMI) estimated. Based on American Diabetes Association (ADA) guidelines [18], symptoms including increased thirst, dry mouth, frequent urination, fatigue, blurred vision, and unintentional weight loss were ascertained through participant self-report and corroborated by clinical assessment where available, with each symptom recorded as a binary outcome. Additionally, we assessed HIV status through a combination of patient self-report and confirmatory diagnostic testing, in accordance with national guidelines. All participants received the same standard TB treatment regimen as per the TB guideline [3]. Approximately 4 mL of venous blood was drawn from enrolled participants in a lavender-top vacutainer, and whole blood was used for baseline hyperglycaemia testing.

At follow-up, two and five months after TB treatment initiation, participants were re-examined for DM symptoms, and blood was drawn for hyperglycaemia testing. Sputum specimens were collected and tested for MTB bacilli as per the TB screening and diagnostic algorithm [3]. TB treatment outcomes were assessed using month-two and month-five sputum MTB test results and were reported as either favourable (smear negative) or unfavourable (smear positive) based on bacteriological treatment response and WHO guidelines [5]. During TB outcome evaluation, we excluded patients who were lost to follow-up.

### Hyperglycaemia screening criteria

Enrolled participants were tested for hyperglycaemia using glycated haemoglobin (HbA1c). Approximately 4 mL of venous blood was collected in a lavender-top vacutainer before TB treatment was initiated and HbA1c levels determined by fluorescence immunoassay technology – sandwich immunodetection method (Finecare™, Guangzhou, P.R. China). At baseline, participants were divided into two: normoglycaemic and hyperglycaemic groups. Those with HbA1c levels ≥5.7% were classified under the hyperglycaemic group, while those presenting with HbA1c levels between 4.0% and 5.6% under the normoglycaemic group. The hyperglycaemic were further classified as having DM (HbA1c ≥ 6.5%) or prediabetes (pre-DM) (HbA1c = 5.7–6.4%) as per the American Diabetes Association (ADA) guideline [18]. Glycaemic status was assessed before initiation of TB treatment, two months after the intensive phase, and 3 months after the start of the continuation treatment phase. The HbA1c trajectory during the initial 2 months of intensive TB treatment and 3 months of continuation treatment phase was defined as follows: Persistent normoglycaemia if HbA1c was between 4.0% and 5.6% at all time points; persistent hyperglycaemia if HbA1c was ≥5.7% at all time points; transient hyperglycaemia if HbA1c was ≥5.7% at only one or two of the time points; and incident hyperglycaemia if the participants were normoglycaemic at baseline and then developed hyperglycaemia at either the 2^nd^ or 3^rd^ time point. After the five-month follow-up period, participants were further categorized into two groups: glycaemic stability if, at the three time points, the participant had HbA1c levels between 4.0 and 5.6%; and glycaemic variability if they had HbA1c levels fluctuating between the normoglycaemic and hyperglycaemic ranges across the three time points.

### Statistical analysis

Data analysis was done using SPSS version 26.0 (IBM Corp., NY, USA) and GraphPad Prism version 9.0 (GraphPad Software, San Diego, CA). The Shapiro-Wilk test was used to test the normality of the sample data set. Continuous variables, such as age and HbA1c, were summarized using median and interquartile ranges (IQRs), while categorical variables, such as sex, education level, BMI, smoking, and alcohol use, were presented as frequencies and percentages. To test for differences between groups, the Mann-Whitney *U* test was used for numerical data, while χ2 or Fisher’s exact test, as appropriate, was used for categorical data. Overall differences in participants’ HbA1c levels across the three study time points were assessed using the Friedman test. This analysis was restricted to participants with complete paired HbA1c measurements at baseline, month 2, and month 5 (complete-case subset), as required for the Friedman test. In observations with significant differences, post hoc pairwise comparison was conducted using the Wilcoxon signed-rank test with Bonferroni correction for multiple testing. The prevalence of normoglycaemia and hyperglycaemia (pre-DM and DM) was estimated with corresponding proportions and 95% confidence intervals. The number needed to screen (NNS), to evaluate the efficiency of screening for hyperglycaemia, prediabetes, and diabetes mellitus among newly diagnosed TB individuals, was calculated as the reciprocal of the point prevalence (proportion) for each parameter.

To robustly model longitudinal associations, a Generalized Estimating Equation (GEE) model with a binomial distribution and log link function to account for within-subject correlation over time was performed. The GEE approach assumed an exchangeable working structure to model the correlation between repeated measures within individuals. Robust (sandwich) standard errors were computed to obtain valid inferences even when the working correlation structure was misspecified. Estimates were reported for the association between hyperglycaemia and clinical symptoms at baseline, two months, and five months, as well as sputum smear conversion and cure results at the second and fifth months of TB treatment. In multivariable GEE analysis, a model was fitted adjusting for a priori covariates selected based on literature and their known associations with glucose metabolism, symptom reporting, and clinical relevance. In symptom overlap, clinical symptoms, including thirst and frequent urination, which are related, were modelled as distinct binary outcomes in separate model analysis, and neither outcome was included as a covariate. However, to assess the robustness of the multivariable GEE model findings given the conceptual symptom overlap, a sensitivity analysis was conducted. Analysis was repeated after excluding participants who reported both symptoms.

To explore and complement the primary regression-based analysis (GEE) and further describe patterns of HbA1c at months 2 and 5, we stratified participants into glycaemic stability and glycaemic variability groups. Within these groups, differences in HbA1c distributions between participants with favourable and unfavourable TB treatment outcomes were assessed using Mann-Whitney *U* test.

Sputum smear conversion was defined as a change from positive to negative status, and the percent conversion calculated as the number of smear-negative cases divided by the total in each group. Statistical significance was set at a p-value of <0.05, and 95% confidence intervals (CI) were reported.

Missing data during the five-month study period was examined for patterns to determine the plausibility of the missing completely at random (MCAR) assumption required for the Generalized Estimating Equation (GEE) analysis. Baseline socio-demographic and clinical characteristics (age, HbA1c, BMI, sex, and marital status) were compared between participants with complete follow-up data and those with incomplete observations. Continuous variables were compared using the Mann-Whitney *U* test, while categorical variables were assessed using the χ2 or Fisher’s exact test, as appropriate. No significant differences in baseline characteristics were observed between the groups, supporting the plausibility of the MCAR assumption. Therefore, the standard GEE model was fitted using all available observations under an available-case approach.

### Ethical statement

The study was reviewed and approved by the University of Nairobi – Kenyatta National Hospital Ethics and Research Committee (P553/06/2023). Permission to conduct the study in Rhodes Chest Clinic and Mbagathi County Hospital was granted by the Nairobi City County, and licensed by the National Commission for Science, Technology & Innovation (NACOSTI/P/23/31666). Participation was voluntary, and written informed consent was obtained from all participants prior to enrolment. Confidentiality was maintained throughout the study by coding personal identifiers.

## Results

### Participant flow and follow-up

**Fig 1:**
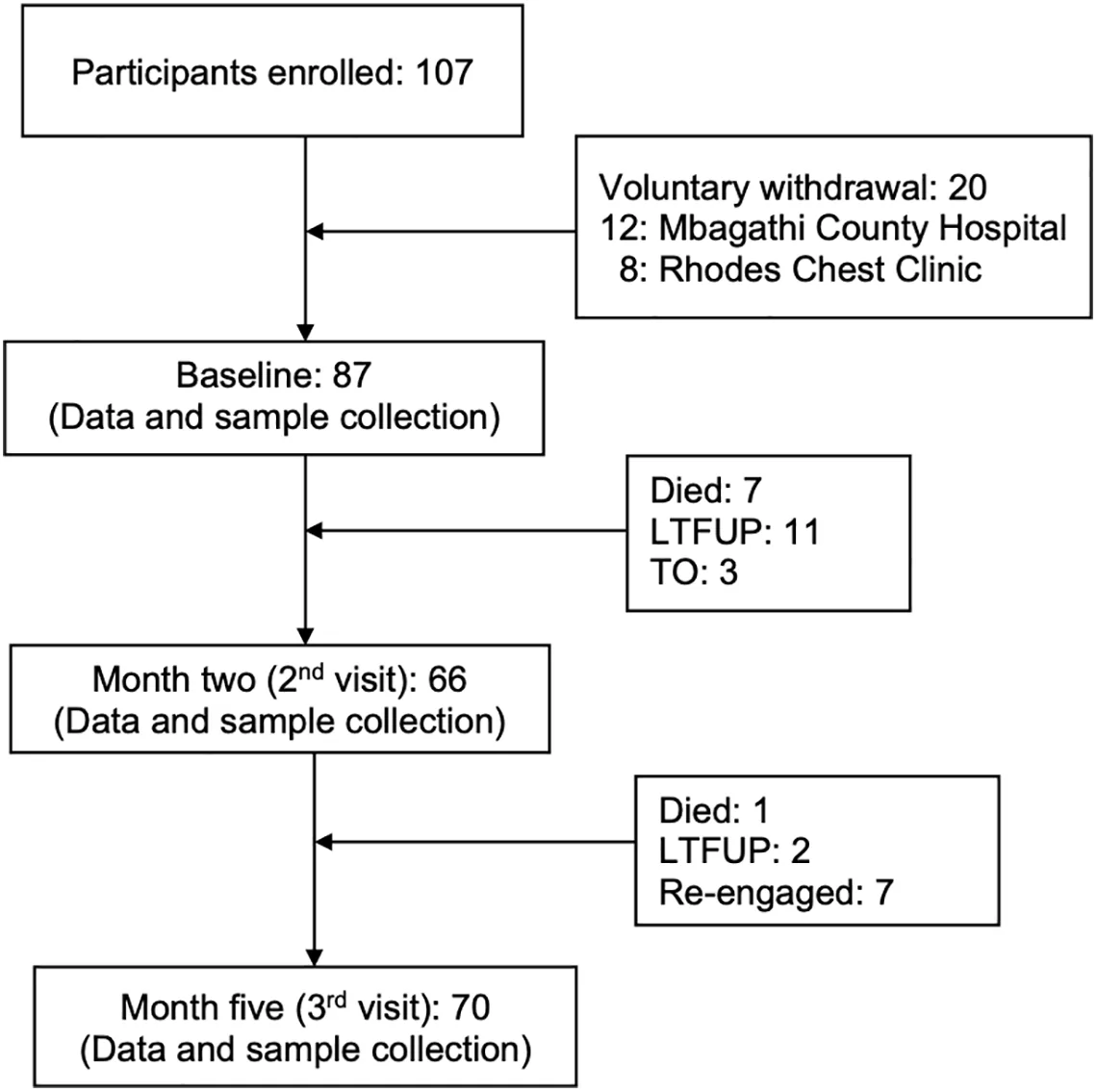
Flow chart of study participants enrolled and followed for five months.

A total of 120 individuals newly diagnosed with PTB were screened for eligibility at Mbagathi County Hospital (50) and Rhodes Chest Clinic (70). Out of these, 107 met the inclusion criteria and were enrolled before TB treatment was initiated. Twenty (20) participants (12 from Mbagathi County Hospital and 8 from Rhodes Chest Clinic) voluntarily withdrew before collecting baseline data and samples after they developed severe complications. Among the 87 participants followed, seven died, three transferred out (TO), and eleven were lost to follow-up (LTFUP) at month two visit. However, among the LTFUP, seven were successfully traced and re-engaged before the month 5 visit, with treatment default primarily attributed to perceived recovery. By month five, two participants were LTFUP, and an additional death was reported (Fig 1).

### Characteristics of the study participants

At baseline, the median age of participants was 37 years (IQR: 29–44), with the majority (67%) aged between 25 and 44. Most participants were male (76%), not married (53%), self-employed (41%), and nearly half had attained secondary education (45%), while 32% had tertiary-level education. Among the participants, 47% had a normal BMI and 70% were non-smokers. Fourteen (16%) participants were HIV-positive, with the majority recruited from Mbagathi County Hospital. All HIV-positive participants were on antiretroviral therapy (ART). At months two and five, follow-up was achieved for 76% (66/87) and 81% (70/87) of participants, respectively. Recruitment was balanced across the two study sites, with 52% (45/87) enrolled at Rhodes Chest Clinic and 48% (42/87) at Mbagathi County Hospital (Table 1).

**Table 1:** Baseline socio-demographic and clinical characteristics of individuals newly diagnosed with pulmonary tuberculosis (N = 87).

Characteristics	n (%)
**Age group (years)**	
18 - 24	9 (10.3)
25 - 44	58 (66.7)
>45	20 (23.0)
**Sex**	
Male	66 (75.9)
Female	21 (24.1)
**Marital status**	
Married	41 (47.1)
Single	46 (52.9)
**Education**	
Primary	20 (23.0)
Secondary	39 (44.8)
Tertiary	28 (32.2)
**Occupation**	
Employed	27 (31.0)
Self employed	36 (41.4)
Unemployed	24 (27.6)
**Body Mass Index (Kg/m**^**2**^)	
<18.4	35 (40.2)
18.5–22.9	41 (47.1)
>23	11 (12.6)
**Smoking**	
Yes	26 (29.9)
No	61 (70.1)
**Alcohol use**	
Yes	52 (59.8)
No	35 (40.2)
**HIV status**	
Positive	14 (16.1)
Negative	73 (83.9)
**Number at follow-up**	
Enrolment	87 (100.0)
Month 2	66 (75.8)
Month 5	70 (80.5)
**Healthcare facility**	
Mbagathi County Hospital	42 (48.3)
Rhodes Chest Clinic	45 (51.7)

### Prevalence of hyperglycaemia

#### Baseline HbA1c.

Among the 87 participants included in the study, 65 had elevated HbA1c levels at enrolment, giving a prevalence of hyperglycaemia of 75% (95% CI: 65–83). Among the 65 hyperglycaemic participants, 39 (45%) had prediabetes (pre-DM) while 26 (30%) had DM.

Median HbA1c levels for the whole study population were 6.0% (inter-quartile range [IQR] 5.6–6.5%. For the hyperglycaemic subgroup, the median HbA1c level was 6.3% (IQR 6.0–6.7), 5.3% (IQR 5.1–5.5) for the normoglycaemic subgroup, 6.0% (IQR 5.8–6.3) for pre-DM, and 6.8% (IQR 6.6–7.2) for DM. Compared to normoglycaemic participants, median HbA1c was significantly higher among the pre-DM (p < 0.001) and the DM (p < 0.001).

Based on the prevalence of hyperglycaemia at enrolment, the number needed to screen (NNS) among the study participants to detect a hyperglycaemic, pre-DM, and DM case before initiating TB treatment was 1.3 (1/0.75), 2.2 (1/0.45), and 3.3 (1/0.3), respectively.

#### Month two HbA1c.

Out of the 66 study participants tested for hyperglycaemia two months after initiation of TB treatment, 36 (55%) were normoglycaemic and 30 (45%) hyperglycaemic. Among the 30 hyperglycaemic participants, HbA1c levels fell within the pre-DM range for 16 (53%) and in the DM level for 14 (47%).

Median HbA1c at month 2 was 5.6% (IQR 5.3–6.3), with 5.3% (IQR 4.8–5.5) for the normoglycaemic group, 6.1% (5.9–6.3) pre-DM, and 7.0% (IQR 6.5–7.2) for DM.

Linking baseline and month 2 HbA1c results for each participant, 25 (38%) had persistent hyperglycaemia, 23 (35%) transient hyperglycaemia, 5 (7%) incident hyperglycaemia, and 13 (20%) maintained a normoglycaemic state at both time points.

#### Month five HbA1c.

Forty-three (61%) of the 70 participants that came back for the fifth month visit had normoglycaemia, and 27 (39%) were hyperglycaemic. Among the hyperglycaemic, 21 (78%) had HbA1c levels that fell within the pre-DM range and 6 (22%) within the DM range.

At month five, the median HbA1c level was 5.4% (IQR 4.9–5.9). After stratification by glycaemic status, median HbA1c was 5.0% (IQR 4.6–5.3) among participants with normoglycaemia, 5.9% (5.8–6.3) in the pre-DM group, and 6.8% (IQR 6.7–7.8) among those with DM.

Linking month 5 with prior time point results, 14 of 62 participants (23%) had persistent hyperglycaemia, 13 (21%) had transient hyperglycaemia, 24 (39%) normoglycaemia, and 11 (18%) incident hyperglycaemia.

#### Overall trend of hyperglycaemia.

The proportion of participants in the DM group decreased by 21% from 30% (26/87) at baseline to 9% (6/70) at month 5, while those in the pre-DM group decreased from 45% (39/87) at baseline to 30% (21/70) at month 5, despite a transient drop to 24% (16/66) at month 2. The proportion of participants with normoglycaemia increased from 25% (22/87) at baseline to 61% (43/70) at month 5, an absolute increase of 36%.

Progressively, the HbA1c levels reduced significantly over the 5-month period (p < 0.001) with a significant difference in median HbA1c levels noted at baseline (n = 87, 6.0%; IQR 5.6–6.5), second month (n = 66, 5.6%; IQR 5.3–6.3), and fifth month (n = 70, 5.4%; IQR 4.9–5.9) (p < 0.001). On further analysis to assess changes in HbA1c across the time points, HbA1c levels differed significantly across the three time points (Friedman test (b_2mo_5mo) χ² (2) = 20.85, p < 0.001, n = 62). On time point post-hoc pairwise comparison analysis, Wilcoxon Signed-Rank test showed significantly lower HbA1c levels at month 2 (5.6%) compared with baseline (6.0%) (Z = −3.06, p = 0.002, r = 0.39); month 5 (5.5%) compared with month 2 (Z = −3.04, p = 0.002, r = 0.39); and month 5 compared with baseline (Z = −4.96, p < 0.001, r = 0.63), with all comparisons remaining statistically significant after Bonferroni correction (adjusted α = 0.017).

At the end of the follow-up period (month 5), glycaemic stability was observed in at least 50% (11/22) of the participants who were normoglycaemic at baseline, while 23% (5/22) experienced high glycaemic variability.

### Symptoms associated with hyperglycaemia in the study population

At baseline, symptoms were compared between 65 hyperglycaemic participants (HbA1c ≥5.7%) and 22 normoglycaemic participants. Among the hyperglycaemic, symptoms were compared between participants presenting with pre-DM (39, HbA1c 5.7–6.4%), DM (26, HbA1c ≥ 6.5%), and those with normoglycaemia. Prediabetic participants reported a higher frequency of urination (56% vs 27%, p = 0.028) and weight loss (64% vs 36%, p = 0.037) compared to the normoglycaemic participants. Similarly, DM participants significantly reported a higher incidence of classic symptoms of hyperglycaemia, such as unintentional weight loss and increased thirst, compared to normoglycaemic participants (p < 0.05) (Table 2).

**Table 2:** Association between glycaemic status and clinical symptoms among study participants before the start of TB treatment.

Symptom		N	Pre-DM N = 39	Normogly N = 22	p value	N	DM N =26	Normogly N = 22	p value
			n (%)	n (%)			n (%)	n (%)	
Increased thirst	Yes	27	20 (51)	7 (32)	0.142	25	18 (69)	7 (32)	**0.010**
	No	34	19 (49)	15 (68)		23	8 (31)	15 (68)	
Frequent urination	Yes	28	22 (56)	6 (27)	**0.028**	26	20 (77)	6 (27)	**0.001**
	No	33	17 (44)	16 (73)		22	6 (23)	16 (73)	
Fatigue	Yes	52	33 (85)	19 (86)	1.000*	41	22 (85)	19 (86)	1.000*
	No	9	6 (15)	3 (14)		7	4 (15)	3 (14)	
Blurred vision	Yes	27	18 (46)	9 (41)	0.692	24	15 (58)	9 (41)	0.247
	No	34	21 (54)	13 (59)		24	11 (42)	13 (59)	
Unintentional weight loss	Yes	33	25 (64)	8 (36)	**0.037**	29	21 (81)	8 (36)	**0.003**
	No	28	14 (36)	14 (64)		19	5 (19)	14 (64)	

DM Diabetes Mellitus; Normogly Normoglycaemia; N total number of participants; Pre-DM prediabetes. Values are presented as n (%) with percentages calculated within columns; p value* Fisher’s exact test

To further understand the clinical relevance of hyperglycaemia during TB disease and treatment, we assessed the longitudinal association between symptoms and HbA1c levels by performing Generalized Estimating Equation (GEE) analysis. The results revealed that hyperglycaemic participants were more likely to experience increased thirst (crude Relative Risk [cRR] 2.28, 95% CI 1.30–3.99, p = 0.004), blurred vision (cRR 1.95, 95% CI 1.18–3.25, p = 0.010), unintentional weight loss (cRR 1.90, 95% CI 1.23–2.95, p = 0.003), and frequent urination (cRR 1.78, 95% CI 1.09–2.92, p = 0.022). Similarly, in multivariable analysis, hyperglycaemia remained associated with increased thirst (adjusted Relative Risk [aRR] 2.37, 95% CI 1.38–4.05, p = 0.002), frequent urination (aRR 1.76, 95% CI 1.12–2.77, p = 0.015), blurred vision (aRR 1.95, 95% CI 1.20–3.18, p = 0.007), and unintentional weight loss (aRR 1.84, 95% CI 1.24–2.75, p = 0.003) compared with normoglycaemic participants. Although not statistically significant, 42% of the participants with hyperglycaemia were more likely to experience fatigue during TB disease and treatment (aRR 1.42, 95% CI 0.96–2.08, p = 0.077) (Table 3). In sensitivity analysis, excluding participants with both thirst and frequent urination, the effect estimates showed minimal change while maintaining statistical significance of association with hyperglycaemia.

**Table 3:** Longitudinal association between hyperglycaemia (HbA1c ≥ 5.7) and clinical symptoms before and during TB treatment.

Dependent variable	N (Baseline/M2/M5/TO	Crude Model^†^		Adjusted Model^§^	
		cRR (95% CI)	P^‡^	aRR (95% CI)	P^‡^
Increased thirst	87/66/70/223	2.28 (1.30 - 3.99)	**0.004**	2.37 (1.38 - 4.05)	**0.002**
Frequent urination		1.78 (1.09 - 2.92)	**0.022**	1.76 (1.12 - 2.77)	**0.015**
Fatigue		1.35 (0.93 - 1.97)	0.118	1.42 (0.96 - 2.08)	0.077
Blurred vision		1.95 (1.18 - 3.25)	**0.010**	1.95 (1.20 - 3.18)	**0.007**
Unintentional weight loss		1.90 (1.23 - 2.95)	**0.004**	1.84 (1.24 - 2.75)	**0.003**

† Unadjusted for confounding factors; Model§ adjusting for age, sex, BMI, HIV, marital status, alcohol use, and occupation; P‡ GEE p-value; cRR crude Relative Risk; aRR adjusted Relative Risk; M2 Month 2; M5 Month 5; TO Total Observations (total repeated measurements across the three time points). N number of participants contributing data at each time point (varies as result of LTFUP, transfer out, and death): Baseline N = 87; M2 N = 66; M5 N = 70; Clinical symptoms modelled as distinct binary outcomes in separate model analysis, and neither outcome was included as a covariate

### Effect of hyperglycaemia on TB treatment outcome

Out of the 87 participants followed during the study period, 14 LTFU/TO and six mortality cases from the hyperglycaemic baseline cohort and one from the normoglycaemic cohort were reported at the second month of TB treatment. Seven re-engagements, 2 LTFU/TO, and an additional death of a participant who had maintained HbA1c levels within the normoglycaemic reference ranges at both baseline and the second month were reported at the fifth month. Although mortality cases were more frequent among hyperglycaemic participants, the association was not statistically significant.

A total of 28 participants had TB-positive sputum results at the end of the study period: 24% (16/66) after the intensive phase and 17% (12/70) at the 2^nd^ follow-up visit. The proportion of positive MTB sputum results at month 2 and month 5 was significantly higher among hyperglycaemic individuals compared to the normoglycaemics (p < 0.05). At both treatment time points, hyperglycaemia was significantly associated with delayed mycobacterial clearance, as indicated by lower sputum conversion and cure rates among hyperglycaemic individuals compared to normoglycaemic participants (60% vs. 89%, p = 0.007 at month 2 and 67% vs. 93%, p = 0.005 at month 5) (Table 4).

**Table 4:** Sputum MTB status among hyperglycaemic and normoglycaemic study participants at months two and five of TB treatment.

Timepoint	Glycaemic status	Sputum results		Total	Treatment Outcome (%)
		Positive	Negative		
Month 2^†^	Hyperglycaemia	12	18	30	60
	Normoglycaemia	4	32	36	89
	Total (N)	16	50	66	76
Month 5^*^	Hyperglycaemia	9	18	27	67
	Normoglycaemia	3	40	43	93
	Total (N)	12	58	70	83

† p = 0.007, month 2 (sputum results versus glycaemic status; χ^2^); * p = 0.005, month 5 (sputum results versus glycaemic status; χ^2^). Treatment outcome – conversion (negative sputum results at month 2/total) and cure (negative sputum results at month 5/total).

**Fig 2:**
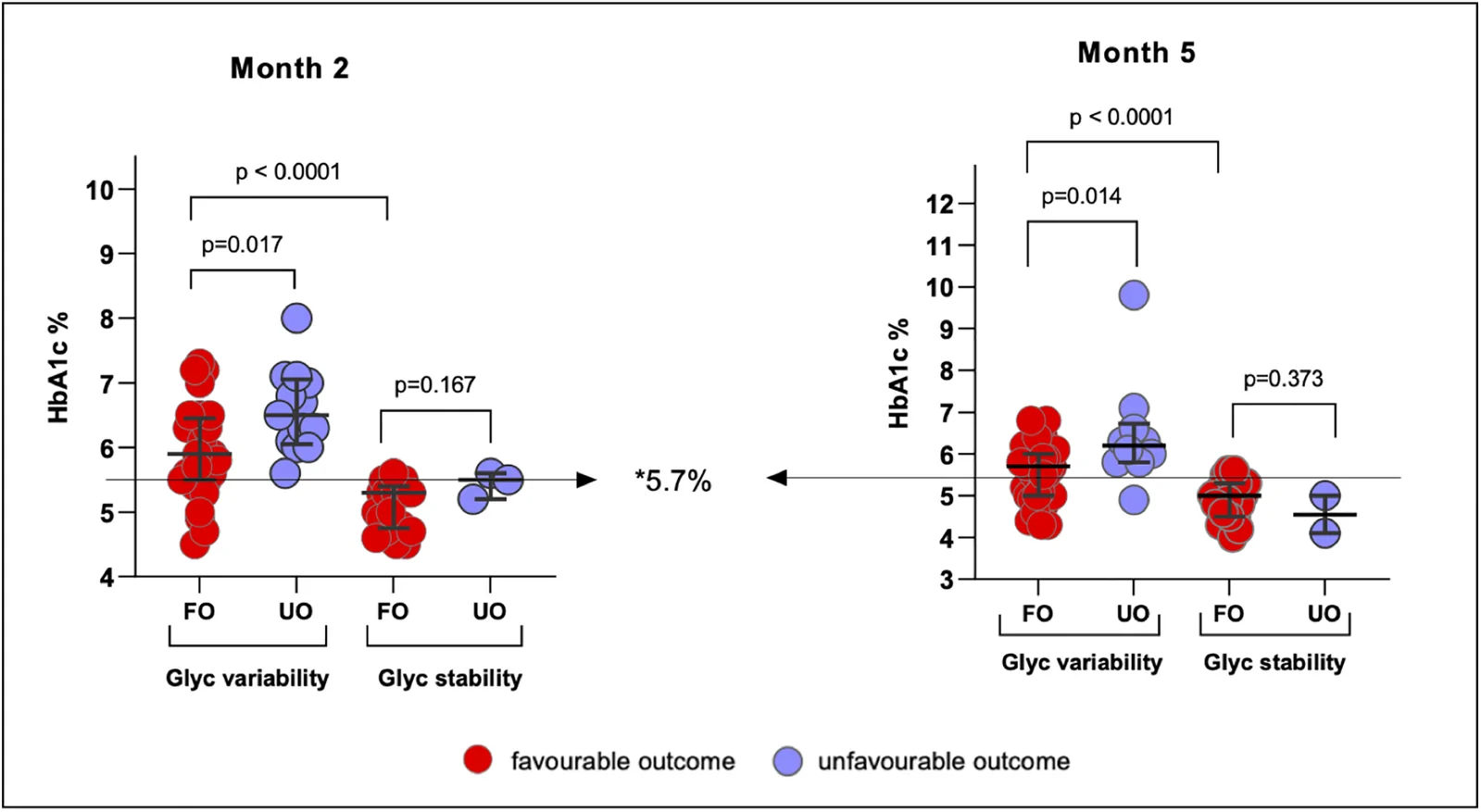
HbA1c levels at Months 2 and 5 stratified by glycaemic status and TB treatment outcomes. Scatter plots of HbA1c (%) stratified by glycaemic pattern (glycaemic variability and glycaemic stability) and TB treatment outcomes at month 2 (left panel) and month 5 (right panel). HbA1c levels were compared between participants with favourable outcomes (FO, red) and unfavourable outcomes (UO, blue) within each glycaemic category using Mann-Whitney test. Horizontal lines represent the median and interquartile range (IQR). The dashed horizontal line at 5.7% indicates the hyperglycaemia threshold.

To further investigate whether hyperglycaemia and related participant characteristics negatively impacted TB treatment outcomes, we performed Generalized Estimating Equation (GEE) analysis. We found that sex, education, occupation, age, and BMI were not independently associated with unfavourable TB treatment outcomes (all p > 0.05). However, hyperglycaemia was associated with a significantly higher risk of unfavourable TB treatment outcome (aRR 4.22, 95% CI 2.09–8.52, p < 0.001), while being married was also independently associated with a higher risk (aRR 2.24, 95% CI 1.04–4.80, p = 0.038). To further describe patterns of glycaemic control, participants were categorized into glycaemic stability and glycaemic variability groups. At month 2, participants in the glycaemic variability group with unfavourable TB treatment outcomes had significantly higher median HbA1c levels compared to those with favourable outcomes (6.5% vs. 5.9%, p = 0.017). In contrast, no significant difference in HbA1c distribution was observed among participants with stable glycaemic profiles (p = 0.167). A similar pattern was observed at month 5, where median HbA1c levels remained significantly higher among participants with unfavourable TB treatment outcomes in the variability group (6.2% vs. 5.7%, p = 0.014), while no significant differences were observed in the stable group (p = 0.373). Moreover, even among the participants who achieved favourable outcomes in the glycaemic variability and in the stability group at months two and five, the HbA1c levels remained significantly different (p < 0.001). Across both time points, a greater proportion of participants with unfavourable TB outcomes had HbA1c levels exceeding the 5.7% hyperglycaemia threshold (Fig 2).

## Discussion

Tuberculosis remains a global public health concern, with associated non-communicable diseases increasing the risk of TB morbidity and mortality. In this study, we aimed to investigate the prevalence and temporal patterns of TB-associated hyperglycaemia and its impact on disease presentation and TB treatment outcomes in newly diagnosed TB individuals with no prior history of diabetes or prediabetes. We found that hyperglycaemia was prevalent in adults newly diagnosed with PTB, persistent during treatment, and was associated with an increased risk of DM-related clinical symptoms and delayed sputum conversion.

The prevalence of hyperglycaemia at enrolment among the newly diagnosed TB participants in our study concurs with findings from other similar studies conducted in Brazil, India, and Bangladesh that reported prevalence ranging from 60% to 75% [19–22]. These findings are substantially high compared to the 16% to 45% reported in different studies carried out in other parts of the world, including China, Peru, and Kenya [1,12,23–25]. Contrary to our findings, a recent meta-analysis reported a pooled hyperglycaemia of 27% in newly diagnosed TB individuals [7]. This high prevalence observed in our study and other similar studies may be attributed to several factors. First, during TB disease, insulin sensitivity is impaired by hormones and cytokines such as cortisol, catecholamines, TNF-α, and IL – 6 secreted as a systemic trigger response to *M.* *tuberculosis* infection, promoting hepatic glucose output [7,26–29]. Second, chronic inflammation noted during TB disease may disrupt pancreatic β-cell function and intensify insulin resistance, even in individuals without a prior history of diabetes, thereby increasing blood glucose levels [8]. Third, in this study, the operational definition of hyperglycaemia included both DM and pre-DM, whereas several previous studies, including meta-analysis, restricted the definition to DM alone [30,31]. Finally, although participants with known DM and pre-DM were excluded in this study, the high prevalence of hyperglycaemia observed at baseline may partly reflect transient stress hyperglycaemia associated with active TB disease. In addition, the findings may be influenced by demographic and epidemiological factors associated with the study population and settings, such as the sedentary lifestyle in urban areas and high background cases of undiagnosed pre-DM in the general population [7,17,18].

The dynamic changes in HbA1c levels noted in this study cohort may reflect the interplay between the host immune response and metabolic stress associated with *M.* *tuberculosis* infection. This temporal pattern, marked by a significant decline of HbA1c levels in the first 2 months of TB treatment, may show transient inflammatory dysglycaemia associated with the acute metabolic and immunological effects of active TB [32,33]. These findings align with observations from several other studies that reported hyperglycaemia detected at baseline was transient and that HbA1c levels declined within the first 2–3 months of TB therapy [32–34]. Similarly, Pardeshi and colleagues, in a study of newly diagnosed TB individuals in India, classified participants into four HbA1c trajectory groups and found that both persistent and transient hyperglycaemia was relatively common during the early months of TB treatment [35]. These findings support the utility of repeated HbA1c testing throughout the course of TB treatment [34,35]. Conversely, a substantial proportion of participants in the present study exhibited persistent hyperglycaemia during TB treatment. This is consistent with findings from earlier studies conducted in Brazil, India, Bangladesh, South Africa, and Kenya, which reported that not all TB-associated hyperglycaemia resolves during TB treatment [7,14,32–35]. Persistent high HbA1c levels may indicate sustained metabolic dysfunction, potentially driven by ongoing inflammatory processes or undiagnosed DM unmasked by active TB. High levels of inflammatory cytokines, such as TNF-α, INF-γ, IL-6, and the IL-17 family, may contribute to hyperglycaemia by inducing insulin resistance, increasing the risk of progression to DM [14,35]. These transient and persistent glycaemic changes may therefore result from the drastic upregulation of the immune system during active TB disease and treatment, revealing an underlying predisposition to DM or worsening existing metabolic abnormalities [14,32,34].

Our study revealed that, over time, hyperglycaemic individuals were more likely to experience classic DM-related symptoms, such as polyuria, polydipsia, and unintentional weight loss, compared to their normoglycaemic counterparts. This association remained stable even after adjusting for age, sex, marital status, HIV status, and BMI, suggesting that these findings are not explained by demographic or clinical confounding. Similarly, the sensitivity analysis showed minimal change in effect estimates, confirming that the observed associations are not driven by closely related symptoms and hyperglycaemia is a key driver of the symptoms reported in this cohort. These findings concur with what has been reported in different studies conducted in Brazil, India, and South Africa, where majority of hyperglycaemic TB individuals exhibited classic DM symptoms before, during, and after TB treatment [6,19–21,32]. Notably, most of the previous studies conducted across the globe investigating the relationship between glycaemic status and TB have focused primarily on DM cohorts, where such symptoms may have been directly associated with DM [12,29,33,36,37]. Observations from the present study, for instance, weight loss during TB treatment, align with those of known cardinal TB symptoms in other studies. Weight change has been majorly used as one of the predictors of treatment response in TB patients. However, in the presence of hyperglycaemia, it may highlight increased morbidity and risk of poor outcomes. The convergence of TB- and DM-related symptoms complicates both diagnosis and clinical monitoring, especially in TB-endemic settings, where routine DM screening among TB patients is not consistently implemented. Moreover, the overlap in clinical manifestations associated with TB and hyperglycaemia is often noted after complications have emerged. Clinically, the magnitude of these risks in the current study, ranging from a 75% to 95% increase in classic DM symptoms in participants with high blood sugar, highlights the substantial physical burden these symptoms place on patients prior to diagnostic intervention. This reinforces the importance of integrated screening and management strategies for TB and associated comorbidity. Moreover, in resource-limited settings, recognizing these critical clinical manifestations could serve as a highly efficient, cost-effective strategy by employing a straightforward symptom checklist during routine triaging to prioritize individuals for diagnostic blood glucose screening, thereby optimizing the use of limited diagnostic reagents.

The potential effect of glycaemic status on TB treatment outcomes in patients with DM during anti-TB treatment has been a major concern among general physicians, endocrinologists, and policymakers. In routine clinical practice, the definition of TB treatment outcome using MTB test results from sputum samples at months 2 and 5 is an important bacteriological indicator of treatment response that is used to monitor TB. Although it does not provide a comprehensive assessment of final TB treatment outcomes, it reflects bacteriological treatment response during follow-up, which may inform the timely implementation of targeted intervention strategies [1,15,16]. In the present study, with a cohort of individuals who were not previously prediabetic or diabetic, we assessed the impact of hyperglycaemia on sputum conversion during TB treatment, given the importance of sputum conversion as a key indicator of TB treatment outcomes. Our findings showed that hyperglycaemia was strongly associated with lower sputum conversion and cure rates at months 2 and 5, respectively. This suggests delayed mycobacterial clearance and, hence, increased risk of persistent infection, prolonged infectivity, and enhanced transmission potential [1,23]. Relating to our findings on the effect of hyperglycaemia on TB treatment outcomes, previous studies in India, China, Brazil, Peru, and South Africa have shown that TB patients presenting with uncontrolled hyperglycaemia have higher Acid-Fast Bacilli (AFB) loads and increased treatment failure rates than the normoglycaemic patients [23,31,35,37,38]. Yanqui and colleagues, in a recent study conducted in China, have also shown that hyperglycaemia doubles the risk of delayed sputum conversion and is associated with unfavourable TB treatment outcomes after the intensive phase of TB treatment [1]. Using the generalized estimating equation analysis, we further confirmed that hyperglycaemia, independently, predicted sputum positivity even after controlling for possible confounding factors. This reinforces the existing evidence that hyperglycaemia is associated with impaired host immune function and delayed mycobacterial clearance, which may partly explain the increased sputum positivity observed among hyperglycaemic participants [1]. Immunologically, hyperglycaemia inhibits the phagocytic function of immune cells such as macrophages, limiting the ability to eliminate the AFB [1,14]. Additionally, hyperglycaemia impairs T-cell development, proliferation, and interferon production, which may further explain the sputum positivity noted in our study and other similar studies [1,14,28,39].

Glycaemic variability and its association with unfavourable TB treatment outcomes were another key observation in this study. By the end of month two and month five, approximately two-thirds of the participants had exhibited high fluctuations in HbA1c levels, where this variability was significantly associated with unfavourable TB treatment outcomes. Importantly, even among those who had favourable TB treatment outcomes, HbA1c levels differed significantly between participants who experienced glycaemic variability and those who maintained stable HbA1c levels. To the best of our knowledge, this is the first study in the region to demonstrate that HbA1c variability in the first months of TB treatment, as opposed to absolute HbA1c, is associated with unfavourable TB treatment outcomes. This aligns with emerging evidence that TB-associated hyperglycaemia in non-DM individuals can impair immune response, promote delayed sputum conversion, and increase the risk of poor clinical and TB treatment outcomes. Findings from several longitudinal studies partly corroborate our findings, suggesting that after bacteriological cure, these patients with poorly controlled glycaemia may experience lasting adverse effects [1,21,23,35,37,40]. Other studies, although focusing on DM patients, have shown that poor glycaemic control during TB treatment predicts relapse, long-term morbidity, and mortality [6,41–43]. Emerging evidence from previous studies suggests that, despite successful TB treatment, a considerable proportion of TB survivors experience persistent pulmonary impairment and cardiovascular dysfunction [44–46]. However, the present study did not assess these outcomes. Future longitudinal studies are needed to determine whether hyperglycaemia during TB treatment influences these long-term health outcomes. Glycaemic variability after TB treatment may partly explain these adverse outcomes, highlighting the need for further studies on the potential link between persistent hyperglycaemia, as an effect of glycaemic variability, and the adverse outcomes among TB survivors. Additionally, in the future, the focus may be to explore the social determinants and unmeasured confounding factors, such as caregiver burden or family-related stress, based on our findings that being married was associated with an increased risk of sputum positivity.

In conclusion, hyperglycaemia was highly prevalent among adults newly diagnosed with PTB, persisted through the fifth month of TB treatment, and was significantly associated with persistent sputum positivity. In addition, high HbA1c variability during TB treatment was associated with unfavourable treatment outcomes, with hyperglycaemic individuals more likely to experience classic DM-related symptoms. Given the rising global rates of DM and the dual burden of TB and DM, especially in TB-endemic countries, these findings support the importance of glycaemic monitoring during TB treatment to identify individuals with persistent dysglycaemia and those at risk of unfavourable TB treatment outcomes. Further longitudinal studies incorporating post-treatment follow-up are needed to determine whether persistent hyperglycaemia resolves after TB treatment or represents underlying metabolic dysfunction requiring ongoing clinical management.

### Strengths and limitations

The major strength of this study is that, to the best of our knowledge, this is the first study in the region and TB-endemic settings with a longitudinal follow-up period beyond the intensive TB treatment phase. In addition, the study focused on individuals not previously diabetic, relative to previous snapshot studies that enrolled DM cohorts. For robust statistical analysis, we used Generalized Estimating Equation (GEE) analysis, an effective statistical method for analysing longitudinal cohort data.

Our study had limitations. First, we did not perform fasting plasma glucose, insulin resistance test, or oral glucose tolerance test, which may have identified cases missed by HbA1c and differentiated transient from persistent hyperglycaemia. However, the longitudinal measurements of HbA1c at multiple time points in this study and adjustment for potential confounders in the statistical analysis strengthen the validity of the findings. In addition, complete blood count parameters, with a focus on haemoglobin (HB), mean corpuscular volume (MCV), mean corpuscular haemoglobin (MCH), mean corpuscular haemoglobin concentration (MCHC), and red cell distribution width (RDW), were assessed to evaluate haematological status and potential anaemia-related influences on HbA1c interpretation. Renal function and insulin resistance markers were not assessed, and the potential impact of insulin resistance and renal impairment on HbA1c measurements cannot be excluded. Second, although we used GEE framework which accommodates missing data, we did not assess the impact of the missing data, comparing complete-case analysis with imputed datasets. Third, our study sample was drawn from urban hospitals, close to where most of the participants lived. This poses geographical and pre-DM and DM-associated lifestyle limitations affecting the generalizability of the results. Additionally, our sample size, though adequate to detect significant associations, and with participants stratified into glycaemic trajectory groups to describe patterns of HbA1c change, may also limit precision and generalizability to other populations, highlighting a need for larger studies. Moreover, the relatively small number of deaths reported in this cohort limited the statistical power to detect differences in mortality between glycaemic groups. Therefore, the absence of statistically significant association and residual confounding related to disease severity and comorbidities cannot be excluded. Fourth, factors such as concomitant medication, diet, disease severity, socioeconomic factors, and adherence to TB medication that were not assessed in this study could partly explain the variability in TB treatment outcomes. Finally, detailed ART regimen data were not collected for HIV-positive participants. Although all HIV-positive participants were receiving ART in accordance with national treatment guidelines, the absence of regimen-specific information precluded assessment of the potential influence of different ART regimens on glycaemic status, HbA1c variability, and TB treatment outcomes. Future studies should incorporate detailed ART treatment data to better evaluate the interaction between HIV treatment, glucose metabolism, and TB outcomes. A further limitation is that treatment response was defined using sputum smear conversion at month 2 and cure at month 5. While these are established indicators of treatment response, reliance on sputum status alone may oversimplify true treatment response by not capturing the broader spectrum of WHO-defined TB treatment outcomes, including treatment completion, failure, loss to follow-up, and death.
